# Metabolic syndrome, insulin resistance and the risk of cardiovascular disease in HIV patients undergoing antiretroviral therapy

**DOI:** 10.1186/1471-2334-13-S1-O30

**Published:** 2013-12-16

**Authors:** Cătălin Tilişcan, Victoria Aramă, Raluca Mihăilescu, Daniela Munteanu, Mihaela Rădulescu, Adriana Hristea, Cristina Popescu, Ruxandra Moroti, Violeta Molagic, Raluca Năstase, Ana Maria Tudor, Mihai Lazăr, Anca-Ruxandra Negru, Irina Lăpădat, Mirela Dinu, Adrian Streinu-Cercel, Daniela Adriana Ion, Sorin Ștefan Aramă

**Affiliations:** 1 Carol Davila University of Medicine and Pharmacy, Bucharest, Romania; 2 National Institute for Infectious Diseases “Prof. Dr. Matei Balş”, Bucharest, Romania

## Background

Most studies published to date have shown an increase in cardiovascular risk (CVR) in patients with HIV infection attributed to both viral activity and antiretroviral therapy (ART). Metabolic syndrome (MS) and insulin resistance syndrome (IR) may represent useful clinical tools for early identification of patients with increased CVR. Our study aimed to evaluate CVR in HIV-infected patients undergoing ART, to identify risk factors for cardiovascular events and to assess the correlations between the presence of MS, IR and CVR.

## Methods

The diagnosis of MS was established using the International Diabetes Federation (IDF) and the American Heart Association/National Heart, Lung, and Blood Institute (AHA/NHLBI) harmonized criteria. Quantitative Insulin Sensitivity Check Index (QUICKI) values were used to quantify IR. CVR was assessed based on Framingham cardiovascular risk score.

## Results

We enrolled 103 patients, including 60 males (58.3%) and 43 females (41.7%). The mean age was 32.3±13.3 years (range: 13-65 years). The median Framingham score was 1.2% (IQR=5.8%). Most patients (81.63%) had a low CVR (below 10%) and 18.37% had Framingham score values above 10%. MS and IR prevalences were 16.9% and 61.2%, respectively. CVR in the general population is primarily dependent on age. This observation was valid for our group: the median age was 24 years in people with low CVR, compared with 50 years for those with Framingham score above 10% (p=0.000). None of the antiretroviral drug classes significantly influenced CVR.

MS patients were approximately five times more likely to have a medium/high CVR (OR 5.3, p=0.000). IR and MS were not significantly correlated (OR 0.8, p=0.642).

## Conclusions

Approximately one fifth of our patients had a medium or high CVR, a significant value, given the young age of those enrolled. Similar to the general population, the most important risk factor for increased CVR was age. The presence of MS and CVR were strongly correlated, suggesting that using MS criteria for increased CVR screening is useful in these patients.

